# Supplementary figures and images for: Relationship between the distribution of vegetation and the environment in the coastal embryo dunes of Jalisco, México
Source: PeerJ. 2022 Mar 2;10:e13015. doi: 10.7717/peerj.13015 (PMC8898010; doi:10.7717/peerj.13015)

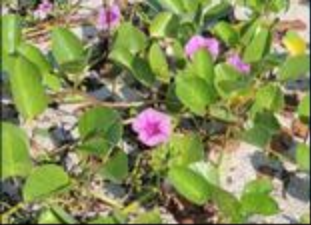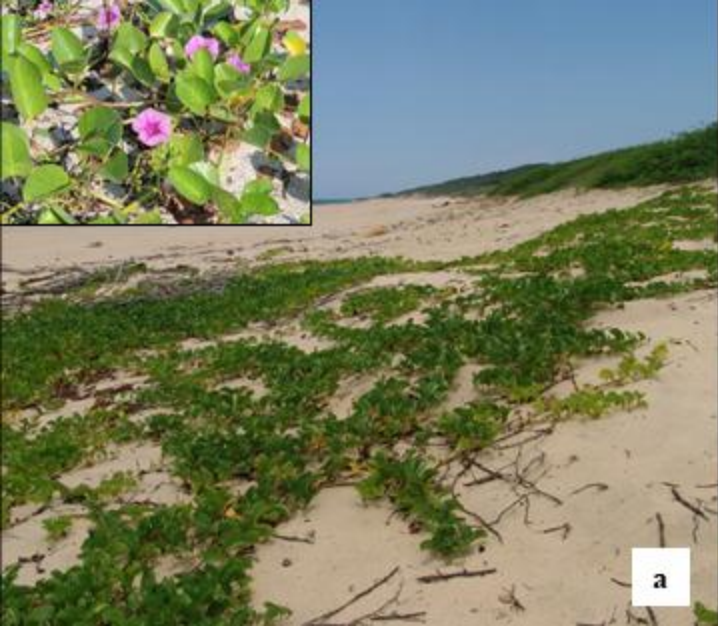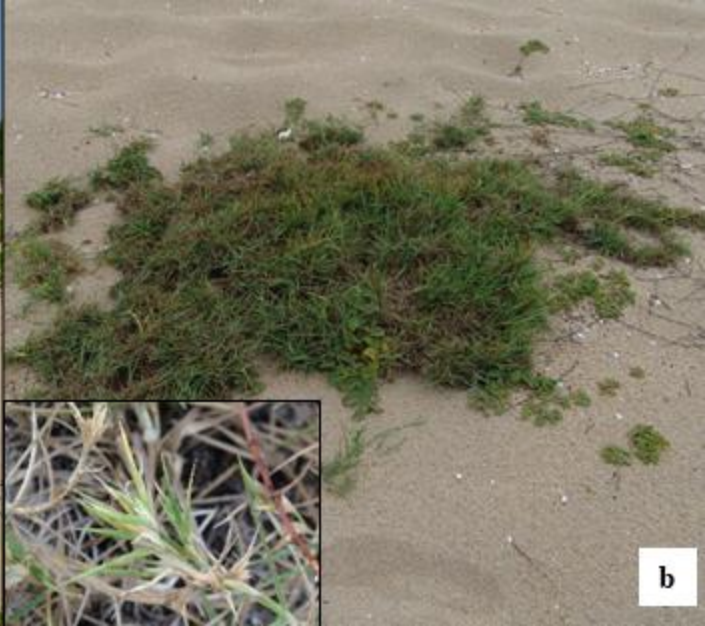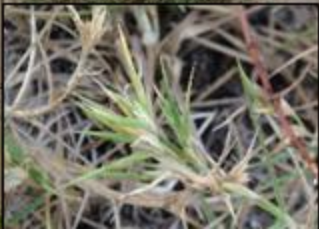

Supplement: Supplemental Information 1 — Photo Credit: Miguel Ángel Macías-Rodríguez [file peerj-10-13015-s001.pdf]

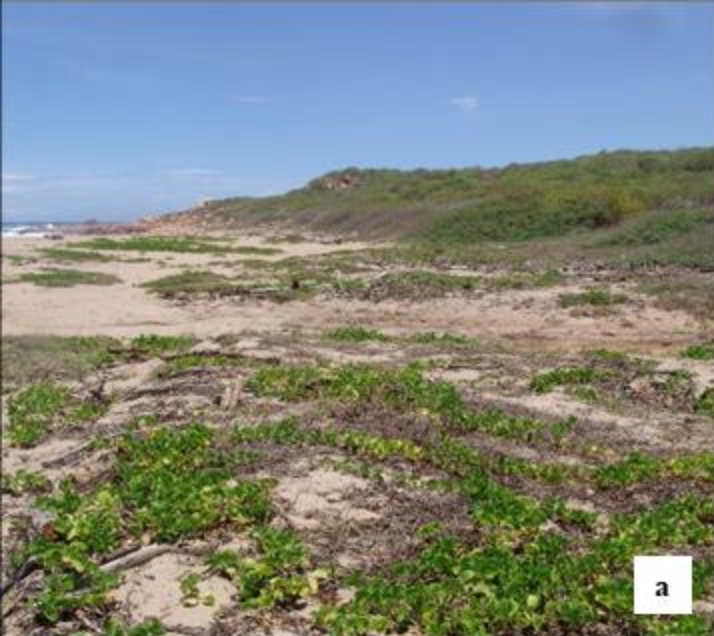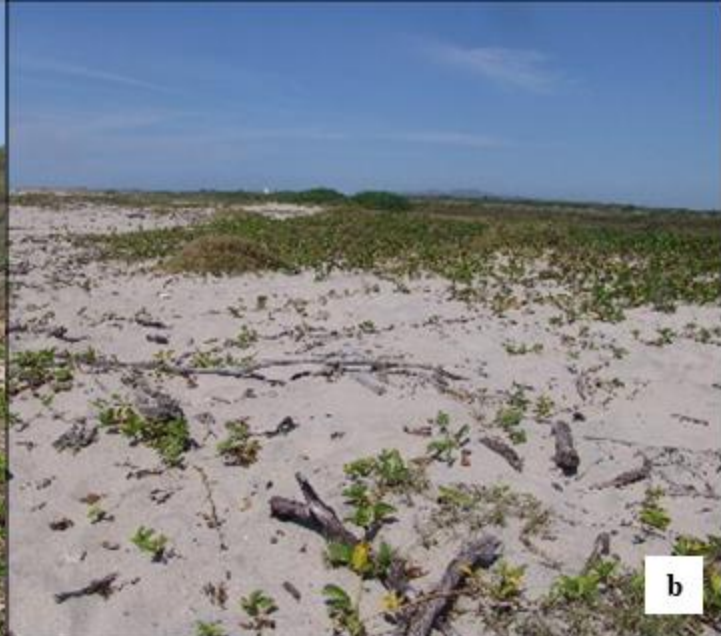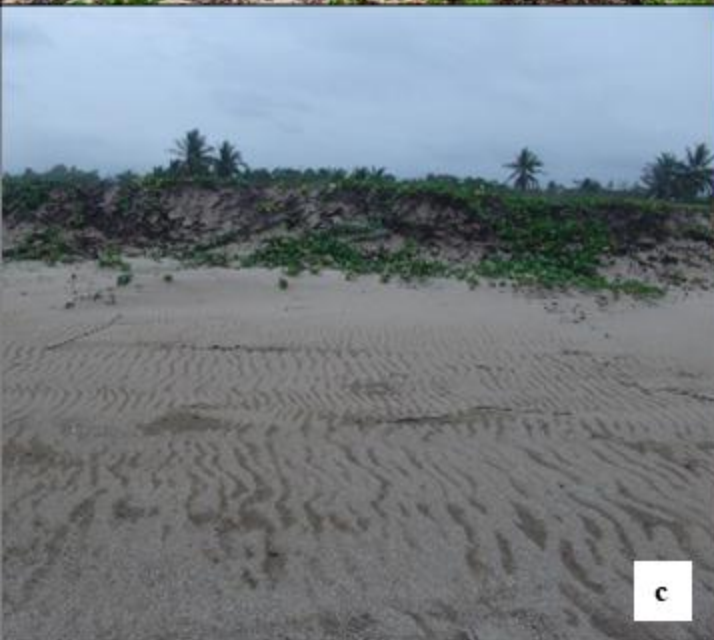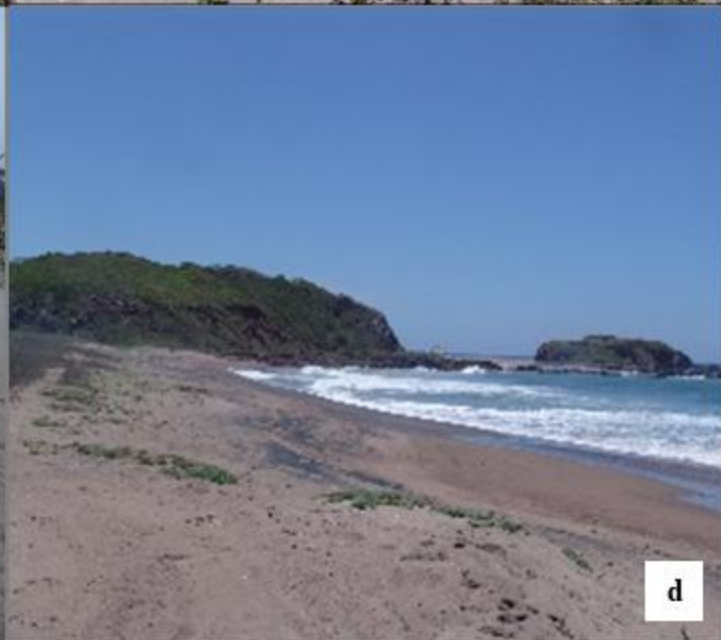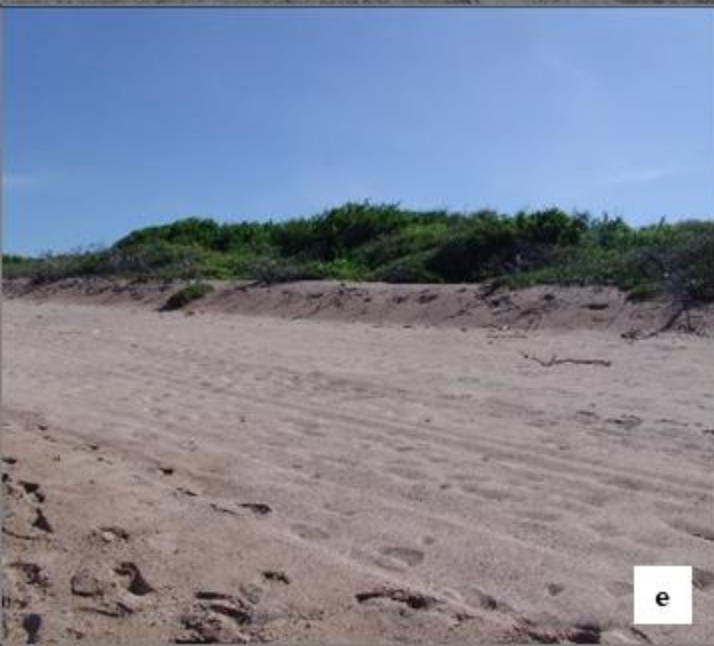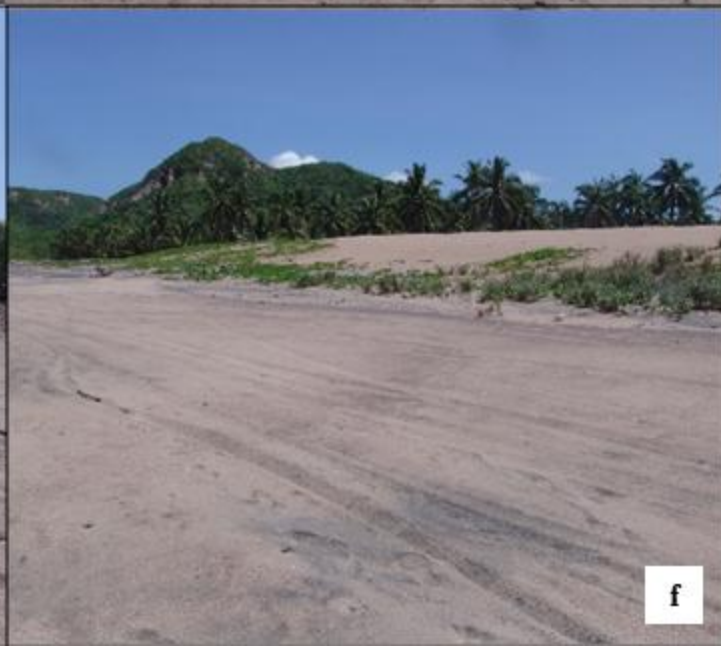

Supplement: Supplemental Information 2 — (a) Las Playitas, (b) El Coco, (c) La Limonera, (d) La Soledad, (e) North Chamela, (f) South El Tecuán. Photo Credit: Héctor Gerardo Frías-Ureña. [file peerj-10-13015-s002.pdf]

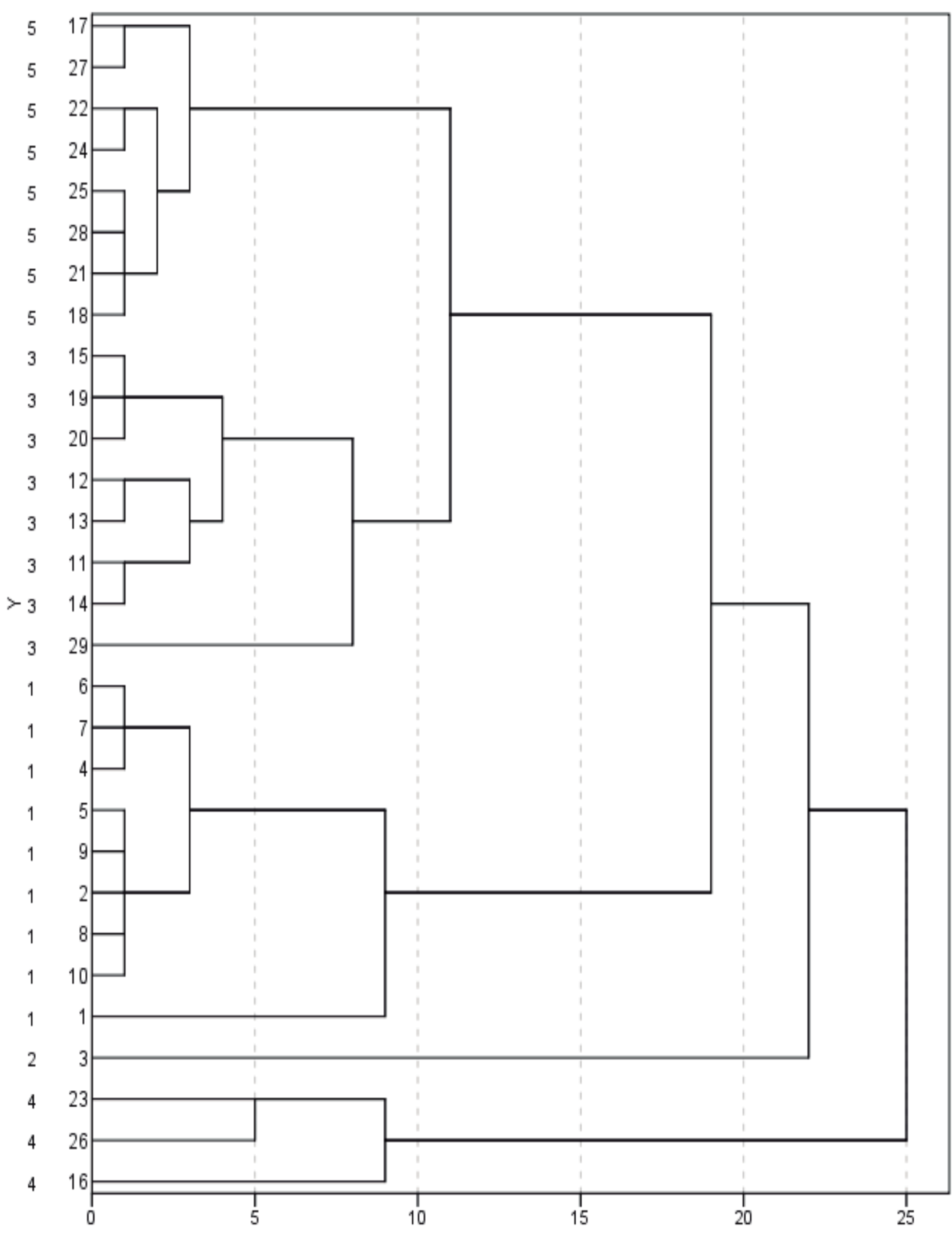

Supplement: Supplemental Information 3 — Re-scaled distance cluster combine. [file peerj-10-13015-s003.pdf]

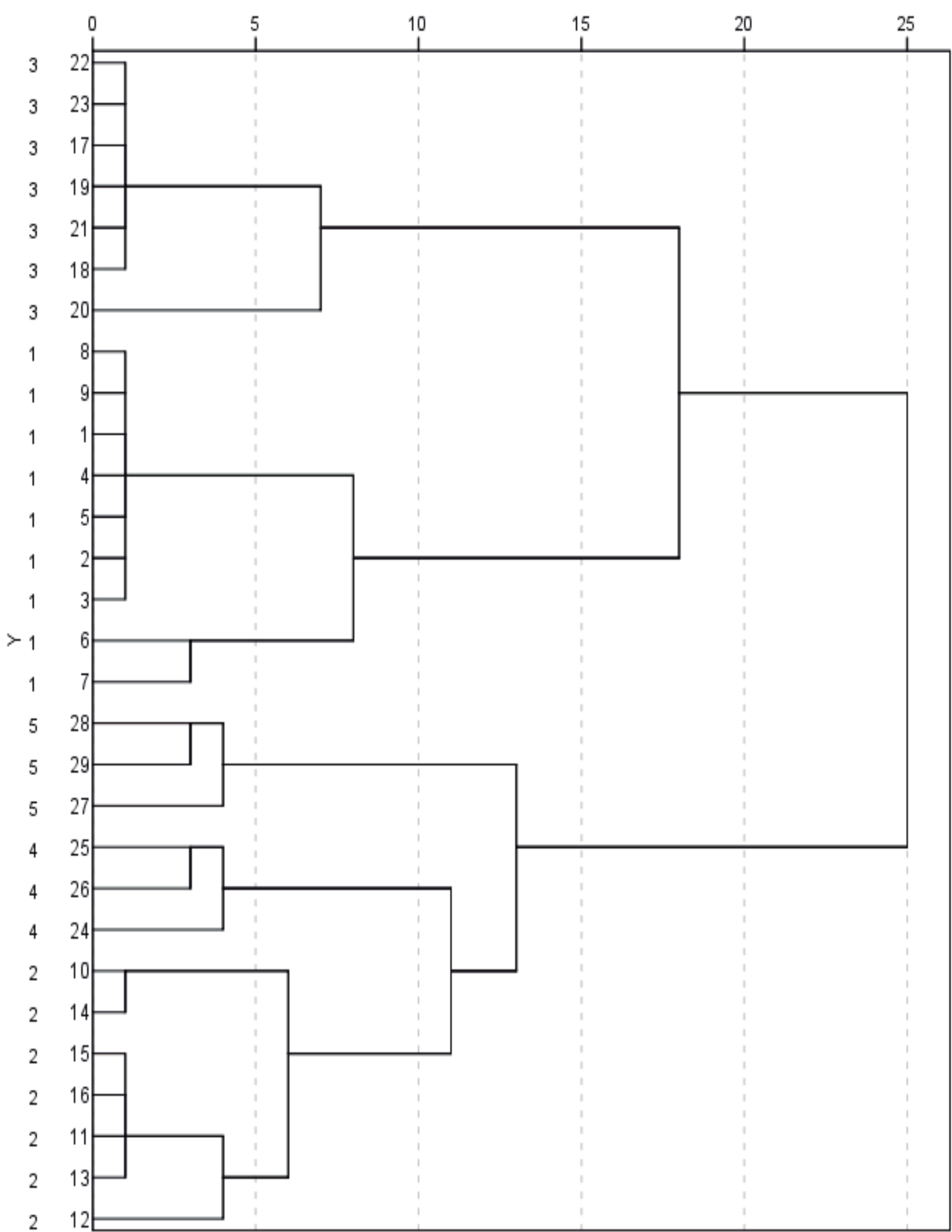

Supplement: Supplemental Information 4 — Re-scaled distance cluster combine. [file peerj-10-13015-s004.pdf]
